# Thromboprophylaxis in lower limb amputation surgery

**DOI:** 10.1590/1677-5449.202301442

**Published:** 2024-08-09

**Authors:** Monique Magnavita Borba da Fonseca Cerqueira, Marcos Arêas Marques, Alcides José Araújo Ribeiro, Daniel Mendes-Pinto, Suzanna Maria Viana Sanches

**Affiliations:** 1 Universidade do Estado da Bahia – UNEB, Salvador, BA, Brasil.; 2 Universidade do Estado do Rio de Janeiro – UERJ, Rio de Janeiro, RJ, Brasil.; 3 Universidade Federal do Estado do Rio de Janeiro – UNIRIO, Rio de Janeiro, RJ, Brasil.; 4 Hospital de Base do Distrito Federal, Brasília, DF, Brasil.; 5 Hospital Felício Rocho, Belo Horizonte, MG, Brasil.; 6 Santa Casa de Misericórdia da Bahia, Salvador, BA, Brasil.

**Keywords:** deep vein thrombosis, amputation, prophylaxis, patient safety, pulmonary embolism, health services research

## Abstract

**Background:**

Lower limb amputation surgery is associated with a high risk of venous thromboembolism. There is evidence that pharmacological thromboprophylaxis is not widely prescribed to patients undergoing this type of procedure.

**Objectives:**

To investigate the profile of the thromboprophylaxis practices of angiologists and vascular surgeons in Brazil during the perioperative period of lower limb amputation surgery and conduct a descriptive analysis of the findings.

**Methods:**

This is a cross-sectional, descriptive study, with simple probabilistic sampling, carried out with angiologists and vascular surgeons working in Brazil. Data were collected through electronic questionnaires, from February to June 2023.

**Results:**

There were 237 respondents, 58.6% of whom conduct thrombotic risk stratification. Of these, 86.3% use the Caprini score. Only 27% of participants stratify patients’ bleeding risk. Low molecular weight heparin is the medication of choice for 85.7% of study participants, 78.9% of whom use a dosage of 40 IU per day. Around 46.8% use direct oral anticoagulants in addition to low molecular weight heparin and rivaroxaban is the drug they most often prescribe (94.6%). A little more than half (51.15%) routinely recommend pharmacological thromboprophylaxis until hospital discharge.

**Conclusions:**

The study revealed the heterogeneous nature of conduct related to prescription of pharmacological thromboprophylaxis, highlighting the need for more studies to support prophylaxis decision-making in this patient population.

## INTRODUCTION

Lower limb (LL) amputations are frequently performed by vascular surgeons because of complications caused by infections, peripheral arterial disease, trauma, or less frequently, cancer.^1^ In Brazil, it is estimated that the incidence of LL amputations is 18.93 per 100,000 inhabitants. From 2010 to 2020, 247,047 admissions related to LL amputation were registered^2^ and, despite improvements in health care, these procedures are still associated with considerable perioperative mortality.^3^

The percentage mortality in the LL amputation population after 30 days varied from 7 to 22%, with higher mortality rates among older patients and those who underwent transfemoral amputation.^4^ A proportion of this mortality can be attributed to thromboembolic complications and so adequate perioperative management of thrombotic risk is essential to reduce morbidity and mortality among amputees.

Individuals who undergo major LL amputations are at high risk of venous thromboembolism (VTE), with incidence ranging from 9.4 to 13.2%.^5-7^ Immobility and surgically induced endothelial venous trauma may be related to the increased thrombotic risk in this population. The risk is not limited to the amputation stump, but can also involve the contralateral limb.^8^ Moreover, comorbidities and risk factors for amputation such as advanced age, smoking, and atherosclerotic disease superimpose conditions that generate a hypercoagulable state and, consequently, increased risk of VTE.^6,7^

Despite the proven efficacy and safety of thromboprophylaxis and its ample publicity over recent years, there are published Brazilian data showing that its use has still not reached the appropriate levels in our country.^9,10^ One of the main barriers to its implementation in hospitals is professionals’ difficulties with systematic thrombotic risk stratification, whether in clinical or surgical patients.^11^

Against this background, the objective of this study was to investigate the profile of the thromboprophylaxis practices of angiologists and vascular surgeons in Brazil during the perioperative period of lower limb amputation surgery and conduct a descriptive analysis of the findings.

## METHODS

This was a cross-sectional descriptive study with simple probabilistic sampling conducted with angiologists and vascular surgeons practicing in Brazil.

From February to June, 2023, electronic questionnaires were sent to all specialists registered with the Brazilian Society of Angiology and Vascular Surgery (SBACV). Additionally, a link to the study was sent to telephone contacts, physicians’ groups on messaging apps (WhatsApp^®^), and posted on the authors’ social networks (Instagram^®^), in order to reach a large sample and include professionals who were not SBACV members. There were no exclusion criteria.

Data were collected using an online form developed with the Google Forms^®^ platform. The survey developed by the authors comprised 10 questions about practices related to LL amputation and thromboprophylaxis, based on references from published literature on the subject. The survey included questions about prescription of pharmacological thromboprophylaxis, thrombotic risk stratification, bleeding risk stratification, and preferred drug class, dosage, and duration used, for patients in the perioperative period of LL amputation surgery.

There are currently approximately 4,000 specialists on the SBACV national registry. Based on this number, it was estimated that a sample of 351 completed questionnaires would be needed to enable statistical inference, calculated for a 5% margin of error and 95% confidence level.

After collection, data were tabulated in a Microsoft Excel^®^ spreadsheet for simple descriptive analysis with absolute and relative frequencies. The project was approved by the Ethics Committee under Ethics Appraisal Submission Certificate number 65867422.0.0000.0057 and decision number 5.820.754.

## RESULTS

**Figure 1:**
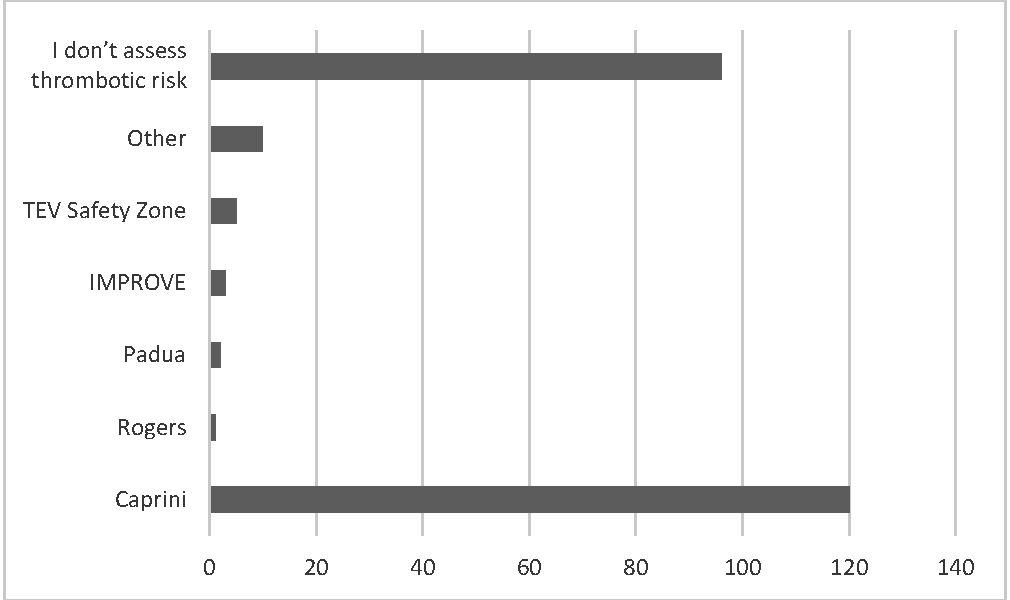
Thrombotic risk assessment models used for lower limb amputation patients.

A total of 237 of the questionnaires sent out were answered in full, with no incompletely or incorrectly answered questionnaires. Inclusions and losses were not calculated. All answered questionnaires were compiled and none were incomplete or had problems. A majority (139 [58.6%]) of the participants conduct thrombotic risk stratification for patients admitted for LL amputation. The Caprini risk assessment model is the most widely used, by 120 (86.3%) participants. The Safety Zone (5 [3.6%]) and Rogers (1 [0.7%]) VTE scores are used by fewer specialists (Figure 1). Although they are not validated for thrombotic risk stratification in surgical patients, the IMPROVE and Padua risk models are used by three (2.2%) and two (1.4%) participants, respectively. Eight respondents (5.8%) use a different score from those listed above.

Just 64 (27%) participants conduct hemorrhagic risk stratification of their patients. The majority (38 [59.4%]) choose the IMPROVE Bleeding Risk Score. Some specialists used the American College of Chest Physicians (10 [15.6%]), HAS-Bleed (6 [9.4%]), and VTE-Bleed (1 [1.6%]) scores, in addition to others not listed (9 [14.1%]).

**Figure 2:**
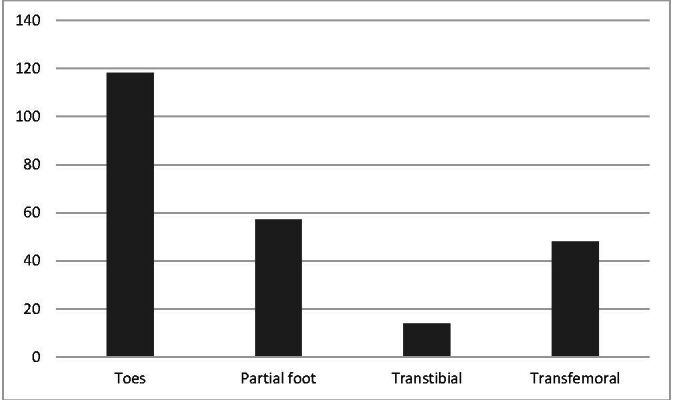
Level of lower limb amputations most often performed.

Around half of the participants (118 [49.8%]) most often perform amputations at the level of the toes. Others most often performed partial foot amputations (57 [24.1%]), transtibial amputations (14 [5.9%]), and transfemoral amputations (48 [20.3%]) (Figure 2). According to 145 (61.2%) specialists, the level of LL amputation has an impact on prescription of thromboprophylaxis.

**Figure 3:**
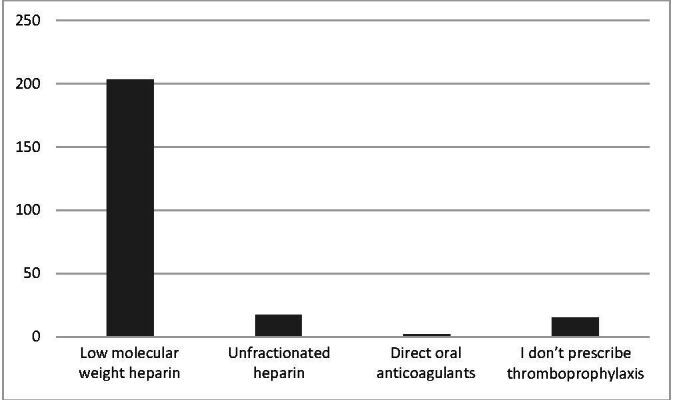
Drug classes most often prescribed for thromboprophylaxis during the perioperative period of lower limb amputation surgery.

Figure 3 illustrates the drug classes most often prescribed for thromboprophylaxis during the perioperative period of surgery for LL amputation, showing that low molecular weight heparin (LMWH) is preferred by 203 (85.7%) of the study participants.

The group that prescribes LMWH most often employs a 40 UI dose, subcutaneously, once a day, which was the preferred option of 187 (78.9%) participants. Twenty participants (8.4%) stated that they use varying dosages, while seven (3%) use a 60 UI dose, subcutaneously, once a day, and four (1.7%) use 20 UI, subcutaneously, once a day. Nineteen (8%) participants stated that they do not use LMWH.

A little less than half (111 [46.8%]) of the respondents use direct oral anticoagulants (DOACs), in addition to LMWH, as part of the pharmacological thromboprophylaxis strategy for LL amputation surgery. Rivaroxaban is the drug of choice of the majority of those who employ drugs in this class (105 [94.6%]), followed by apixaban (5 [4.5%]) and edoxaban (1 [0.9%]). None of the participants endorsed dabigatran.

**Figure 4:**
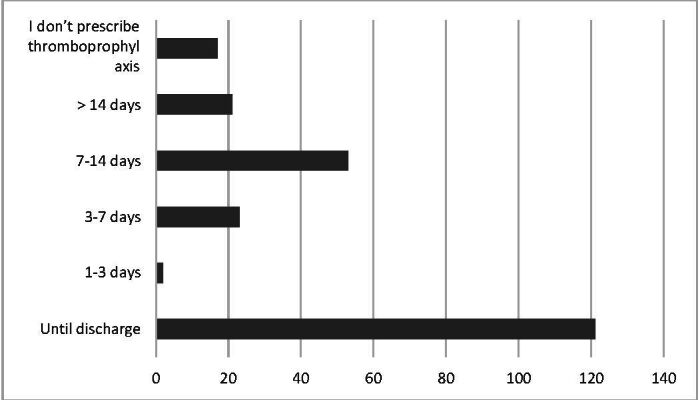
Duration of pharmacological thromboprophylaxis prescription during the perioperative period of lower limb amputation surgery.

With regard to the duration of pharmacological thromboprophylaxis, Figure 4 illustrates the routine prescribed by a little more than half of the specialists up to the point of hospital discharge.

## DISCUSSION

The wider group of patients who undergo LL amputation encompasses several subpopulations with differing levels of thrombotic risk, which varies depending on individual factors – advanced age, obesity, preoperative infection, long-term peripheral arterial disease, and identifiable hypercoagulability status – and also on factors relating to the procedure, primarily duration of surgery and level of amputation.^12,13^ It is essential that VTE risk stratification is performed using a model validated specifically for the subpopulation concerned, employed systematically at each of the major stages of care: hospital admission, transfer between department, and hospital discharge. It should be emphasized that the discharge assessment, in particular, is of great importance in those patients who still have VTE risk factors, such as prolonged immobility.^14^

To date, there is no specific model for assessment of thrombotic risk for these patients. The Caprini score was originally validated for general abdominal and pelvic, vascular, bariatric, and reconstructive plastic surgery and is currently the best tool for this purpose, even though it does not address the specifics of this subpopulation.^15^

The data collected in this study showed that a little more than half of the interviewees stratify their patients’ thrombotic risk and that the great majority use the Caprini score for this purpose, illustrating that its use has been incorporated from other types of vascular surgery. While few, it should be noted that some specialists use scores that have not been validated for surgical patients, such as the IMPROVE and Pádua scores.

In contrast, it was found that only just over 1/4 of the interviewees habitually assess their patients’ hemorrhagic risk, which may indicate that there is a gap in their knowledge of information supporting this practice. In vascular surgery, pharmacological thromboprophylaxis is associated with different levels of bleeding risk, which vary depending on the procedure. However, in general, it is known that this risk is around 2% or more for minor episodes of bleeding and less than 1% for major bleeding.^14,15^ In the absence of a specific model for assessment of bleeding risk, since this is a subject that has been studied little in the non-orthopedic surgical population, the recommendation is to check for presence of comorbidities associated with hemorrhagic events such as advanced age, thrombocytopenia, anemia, and renal or hepatic dysfunction, among others.^14^

The pharmacological thromboprophylaxis drug class of choice among the participants in this study was LMWH, and the regimen most often reported was enoxaparin at a dosage of 40 UI, subcutaneously, once a day. This conduct is in line with the prophylaxis recommendations for the majority of the surgical population.^16^

Among those who choose to use DOACs, the drug of choice was rivaroxaban. This coincides with the findings of a study of thromboprophylaxis for LL varicose vein surgery in Brazil.^17^ The choice of rivaroxaban rather than other DOACs may be influenced by several factors, including cost, safety profile, and posological convenience. It is also possible that the specialists prefer rivaroxaban because they are more familiar with it, since it has been studied most and has been on the market the longest. It is important to point out that edoxaban was also mentioned, even though its use for primary prophylaxis was not supported by pivotal studies.

The duration of use of pharmacological thromboprophylaxis was highly variable, but there was a discrete predominance of only prescribing during the hospital stay. It was not possible to establish an association between individual thrombotic risk and the duration for which pharmacological thromboprophylaxis was prescribed. It is known that the duration of anticoagulation prophylactic should be based on the Caprini score, so that only patients classified as moderate risk (Caprini score 3 or 4) should be given prophylaxis exclusively while in hospital.^18,19^ Although the risk of VTE after hospital discharge is well recognized among high-risk patients, studies demonstrate that extended prophylaxis is still underutilized.^12,15^

This survey was subject to certain limitations. The number of recipients who received the electronic questionnaire cannot be determined exactly since it was sent out via the official SBACV communication channels and also publicized via the researchers’ social networks, resulting in some professionals receiving it multiple times. Moreover, no detailed information was collected on the profile of the services where the specialists practice, but it is probable that the ways in which they prescribe pharmacological thromboprophylaxis varies from region to region in Brazil.

Notwithstanding these limitations, this initial survey of the conduct of angiologists and vascular surgeons practicing in Brazil suggests a need for development and implementation of clinical protocols and therapeutic guidelines for prevention in a population that can be at high risk for VTE occurrence.

## CONCLUSIONS

This study revealed the heterogeneous nature of conduct related to prescription of pharmacological thromboprophylaxis for patients undergoing LL amputation procedures, especially with regard to the tools used for thrombotic risk stratification and the duration of maintenance of prophylactic anticoagulation. Moreover, bleeding risk was not assessed by the majority of interviewees. These findings reflect the need for more studies to correctly stratify these patients, with the objective of supporting decision making related to prophylaxis in this population.
