# Supplementary material for: Thromboprophylaxis in lower limb amputation surgery
Source: J Vasc Bras. 2024 Aug 9;23:e20230144. doi: 10.1590/1677-5449.202301442 (PMC11404779; doi:10.1590/1677-5449.202301442)
Supplement: Translation

# Translation: Tromboprofilaxia na cirurgia de amputação de membros inferiores

**DOI:** 10.1590/1677-5449.202301441

**Authors:** Monique Magnavita Borba da Fonseca Cerqueira, Marcos Arêas Marques, Alcides José Araújo Ribeiro, Daniel Mendes-Pinto, Suzanna Maria Viana Sanches

**Affiliations:** 1 Universidade do Estado da Bahia – UNEB, Salvador, BA, Brasil.; 2 Universidade do Estado do Rio de Janeiro – UERJ, Rio de Janeiro, RJ, Brasil.; 3 Universidade Federal do Estado do Rio de Janeiro – UNIRIO, Rio de Janeiro, RJ, Brasil.; 4 Hospital de Base do Distrito Federal, Brasília, DF, Brasil.; 5 Hospital Felício Rocho, Belo Horizonte, MG, Brasil.; 6 Santa Casa de Misericórdia da Bahia, Salvador, BA, Brasil.

**Keywords:** trombose venosa profunda, amputação, profilaxia, segurança do paciente, embolia pulmonar, pesquisa sobre serviços de saúde

## Abstract

**Contexto:**

As cirurgias de amputação de membros inferiores são associadas a alto risco de tromboembolismo venoso. Existem evidências de que a farmacoprofilaxia não é amplamente prescrita para pacientes submetidos a esse tipo de procedimento.

**Objetivos:**

Conhecer o perfil e realizar a análise descritiva das práticas de tromboprofilaxia no perioperatório das cirurgias de amputação de membros inferiores feitas por angiologistas e cirurgiões vasculares no Brasil.

**Métodos:**

Tratou-se de um estudo transversal, descritivo e de amostragem probabilística simples, realizado entre angiologistas e cirurgiões vasculares atuantes no Brasil. Os dados foram coletados por meio de questionários eletrônicos no período de fevereiro a junho de 2023.

**Resultados:**

Entre os 237 respondentes, 58,6% realizavam a estratificação do risco trombótico, e 86,3% deles utilizavam o escore de Caprini. Apenas 27% dos participantes realizavam a estratificação do risco hemorrágico do paciente. A heparina de baixo peso molecular é a medicação de preferência de 85,7% dos participantes, sendo a dose de 40 UI por dia a escolha de 78,9% deles. Cerca de 46,8% utilizavam, além da heparina, anticoagulantes orais diretos, e, entre eles, a rivaroxabana é a droga mais prescrita (94,6%). Um pouco mais da metade (51,15%) tem como rotina a indicação da farmacoprofilaxia até o momento da alta hospitalar.

**Conclusões:**

O estudo expôs a heterogeneidade nas condutas relacionadas à prescrição de tromboprofilaxia farmacológica, evidenciando a necessidade de mais estudos para dar respaldo à tomada de decisão relacionada à profilaxia nessa população de pacientes.

## INTRODUÇÃO

As amputações de membros inferiores (MMII) são procedimentos frequentemente realizados por cirurgiões vasculares como consequência de complicações causadas por infecção, doença arterial periférica, trauma ou, menos frequentemente, neoplasia^1^ . No Brasil, estima-se que a incidência de amputações de MMII seja de 18,93 por 100.000 habitantes. No período de 2010 a 2020, foram registradas 247.047 internações relacionadas a amputação/desarticulação de MMII^2^ , e, apesar das melhorias na assistência à saúde, ainda existe uma mortalidade perioperatória substancial associada a esses procedimentos^3^ .

A porcentagem de óbitos após 30 dias na população submetida à amputação de MMII variou de 7 a 22%, sendo maiores as taxas de mortalidade entre os mais velhos e entre aqueles submetidos à amputação transfemoral^4^ . Parte dessa mortalidade pode ser atribuída a complicações tromboembólicas, de modo que o manejo perioperatório adequado do risco trombótico é essencial para a redução da morbimortalidade entre amputados.

Indivíduos submetidos a amputações maiores de MMII têm alto risco de tromboembolismo venoso (TEV), com incidência variando entre 9,4 e 13,2%^5-7^ . Imobilidade e trauma endotelial venoso cirurgicamente induzido podem estar relacionados ao aumento do risco trombótico nessa população. O risco não se limita ao coto amputado e pode envolver o membro contralateral^8^ . Além disso, comorbidades e fatores de risco para amputação como idade avançada, tabagismo e doença aterosclerótica se superpõem às condições que geram um estado de hipercoagulabilidade e, consequentemente, de aumento de risco de TEV^6,7^ .

Embora a tromboprofilaxia possua eficácia e segurança comprovadas e tenha sido amplamente difundida nos últimos anos, há dados na literatura nacional evidenciando que, em nosso meio, ela ainda não atingiu os níveis desejados de utilização^9,10^ . Entre as principais barreiras para a sua implementação nos hospitais, estão a dificuldade dos profissionais em sistematizar a estratificação de risco trombótico, tanto em pacientes clínicos quanto cirúrgicos^11^ .

Diante desse cenário, o objetivo desta pesquisa foi conhecer o perfil e realizar a análise descritiva das práticas de tromboprofilaxia no perioperatório de cirurgias de amputação de MMII feitas por angiologistas e cirurgiões vasculares no Brasil.

## MÉTODOS

Tratou-se de um estudo transversal, descritivo e de amostragem probabilística simples, realizado entre angiologistas e cirurgiões vasculares atuantes no Brasil.

No período de fevereiro a junho de 2023, foram enviados questionários eletrônicos a todos os especialistas associados à Sociedade Brasileira de Angiologia e de Cirurgia Vascular (SBACV). Adicionalmente, o *link* da pesquisa foi divulgado por meio de contatos telefônicos, grupos de médicos em aplicativos de mensagens (WhatsApp^®^) e redes sociais dos autores da pesquisa (Instagram^®^), de forma a abranger uma amostragem ampla e incluir profissionais que não fossem membros da SBACV. Não houve critérios de exclusão.

Os dados foram coletados por meio de formulário on-line, utilizando-se a ferramenta Google Formulários^®^. O formulário elaborado pelos autores era composto por 10 perguntas sobre práticas relativas à amputação de MMII e tromboprofilaxia, utilizando como referência informações da literatura publicadas sobre o tema. Fizeram parte do formulário questões sobre prescrição de farmacoprofilaxia, estratificação de risco trombótico, estratificação de risco de sangramento, além de classe medicamentosa de preferência, dose e tempo de utilização do fármaco entre os pacientes que se encontravam no perioperatório de cirurgia de amputação de MMII.

Atualmente, aproximadamente 4.000 especialistas estão registrados no cadastro nacional da SBACV. Com base nesse número, calculou-se que uma amostra de 351 questionários respondidos seria necessária para permitir inferências estatísticas, levando em conta uma margem de erro de 5% e grau de confiança de 95%.

Após a coleta, os dados foram tabulados em planilha do Microsoft Excel^®^ para análise descritiva simples, com frequências absolutas e relativas. O projeto obteve aprovação do comitê de ética sob o Certificado de Apresentação de Apreciação Ética n.º 65867422.0.0000.0057 e parecer n.º 5.820.754.

## RESULTADOS

Entre os questionários enviados, 237 foram respondidos integralmente e compilados sem registro de preenchimento incompleto ou com erros. Não foram contabilizadas inclusões ou perdas. Todos os questionários com alguma resposta foram compilados, e não houve preenchimento incompleto ou com problemas. A maioria (139 [58,6%]) dos participantes realiza estratificação do risco trombótico do paciente internado para amputação de MMII. O modelo de acesso de risco de Caprini é o mais utilizado, sendo usado por 120 (86,3%) participantes. Já os escores TEV Safety Zone (5 [3,6%]) e Rogers (1 [0,7%]) são escolhidos com menos frequência entre os especialistas (Figura 1). Embora não sejam validados para estratificação de risco trombótico entre pacientes cirúrgicos, os modelos de risco IMPROVE e Pádua são a opção de três (2,2%) e dois (1,4%) participantes, respectivamente. Oito respondentes (5,8%) utilizam outro escore além dos citados.

**Figura 1:** Modelo de acesso de risco trombótico utilizado para a amputação de membros inferiores.

Apenas 64 (27%) participantes realizam a estratificação do risco hemorrágico dos pacientes. Para a maior parte (38 [59,4%]), a escolha preferencial é o IMPROVE Bleeding Risk Score. Os escores do American College of Chest Physicians (10 [15,6%]), HAS-Bleed (6 [9,4%]), VTE-Bleed (1 [1,6%]) e outro além dos listados (9 [14,1%]) também são utilizados pelos especialistas.

Cerca de metade dos participantes (118 [49,8%]) realiza mais frequentemente amputações ao nível dos pododáctilos. Os demais têm como nível de amputação mais habitualmente indicado as amputações parciais de pé (57 [24,1%]), as transtibiais (14 [5,9%]) e as transfemorais (48 [20,3%]) (Figura 2). Para 145 (61,2%) especialistas, o nível de amputação de MMII impacta na prescrição de tromboprofilaxia.

**Figura 2:** Nível de amputação de membros inferiores mais frequentemente realizada.

A Figura 3 ilustra a classe medicamentosa mais comumente prescrita para tromboprofilaxia no perioperatório de cirurgia de amputação de MMII, evidenciando que a heparina de baixo peso molecular (HBPM) é a preferência de 203 (85,7%) participantes da pesquisa.

**Figura 3:** Classe medicamentosa mais prescrita para tromboprofilaxia no perioperatório de cirurgia de amputação de membros inferiores.

O grupo de prescritores de HBPM utilizou com mais frequência a dose de 40 UI, subcutânea e uma vez ao dia, sendo a escolha de 187 (78,9%) deles. Vinte participantes (8,4%) referiram usar doses variáveis, enquanto sete (3%) utilizavam a dose de 60 UI, subcutânea e uma vez ao dia, e quatro (1,7%) utilizavam 20 UI, subcutânea e uma vez ao dia. Dezenove (8%) participantes relataram não utilizar a HBPM.

Um pouco menos da metade (111 [46,8%]) dos respondentes utiliza os anticoagulantes orais diretos (DOACs), além da HBPM, como estratégia de farmacoprofilaxia na cirurgia de amputação dos MMII. Entre aqueles que utilizam essa classe medicamentosa, a rivaroxabana é a droga de escolha da maioria (105 [94,6%]), seguida da apixabana (5 [4,5%]) e da edoxabana (1 [0,9%]). A dabigatrana não foi registrada como opção entre os participantes.

No que diz respeito ao tempo de farmacoprofilaxia prescrito pelos especialistas, a Figura 4 ilustra que a rotina entre um pouco mais da metade é a indicação até o momento da alta hospitalar.

**Figura 4:** Tempo de prescrição da farmacoprofilaxia no perioperatório de cirurgia de amputação de membros inferiores.

## DISCUSSÃO

O grupo de pacientes submetido a amputação de MMII compreende subpopulações com risco trombótico diverso, que varia conforme fatores individuais – idade avançada, obesidade, infecção pré-operatória, doença arterial periférica de longa duração e condição de hipercoagulabilidade identificável – e fatores relativos ao procedimento, sobretudo tempo cirúrgico e nível de amputação^12,13^ . Na estratificação de risco de TEV, é fundamental a utilização de um modelo validado especificamente para a população em questão, aplicado de maneira sistemática nas principais etapas de assistência: admissão hospitalar, transição entre setores e alta hospitalar. Destaca-se que a avaliação na alta, particularmente, reveste-se de grande importância nos pacientes que mantêm fatores de risco para TEV, como a imobilidade prolongada^14^ .

Até o momento, não há um modelo de acesso de risco trombótico específico para esses pacientes. O escore de Caprini – originalmente validado para cirurgias geral abdominal e pélvica, vascular, bariátrica e plástica reconstrutora – é a melhor ferramenta no contexto atual para esse fim, embora não contemple as especificidades dessa população^15^ .

Os dados obtidos neste estudo evidenciam que pouco mais da metade dos entrevistados estratifica o risco trombótico de seus pacientes, e, para esse fim, a grande maioria utiliza o escore de Caprini, refletindo a incorporação do seu uso a partir do cenário das demais cirurgias vasculares. Apesar da baixa representatividade, vale ressaltar que escores não validados para pacientes cirúrgicos, como IMPROVE e Pádua, são utilizados por alguns especialistas.

Em contrapartida, percebe-se que apenas um pouco mais de 1/4 dos entrevistados têm o hábito de avaliar o risco hemorrágico dos pacientes, o que pode refletir a existência de uma lacuna de informações para respaldar essa prática. A farmacoprofilaxia em cirurgia vascular está associada a diferentes graus de risco de sangramento, que variam conforme o procedimento. Sabe-se, no entanto, que, em geral, esse risco gira em torno de 2% ou mais para episódios de sangramento menor e em menos de 1% para sangramento maior^14,15^ . Na ausência de um modelo de acesso de risco de sangramento específico, uma vez que se trata de um assunto pouco estudado na população cirúrgica não ortopédica, recomenda-se avaliar a existência de comorbidades que estejam associadas a eventos hemorrágicos como idade avançada, plaquetopenia, anemia, disfunção renal ou hepática, entre outras^14^ .

A classe farmacológica de eleição para farmacoprofilaxia entre os participantes deste estudo foi a HBPM, sendo a enoxaparina na dose de 40 UI, subcutânea e uma vez ao dia a droga mais utilizada. Essa conduta está em conformidade com as recomendações de profilaxia para a maioria da população cirúrgica^16^ .

Entre os que optaram pelo uso dos DOACs, a droga de escolha foi a rivaroxabana. Esse dado coincide com os achados de um estudo sobre tromboprofilaxia na cirurgia de varizes dos MMII no Brasil^17^ . A escolha da rivaroxabana em comparação com outros DOACs pode ter sido influenciada por diversos fatores, incluindo custo, perfil de segurança e comodidade posológica. Além disso, é possível que os especialistas a prefiram devido à sua maior familiaridade com a droga, decorrente do fato ter sido a mais estudada e de estar disponível no mercado há mais tempo. É importante pontuar que a edoxabana foi citada como opção, apesar do seu uso para profilaxia primária não ser respaldado por estudos pivotais da droga.

O tempo de uso da farmacoprofilaxia foi bastante variável, mas houve uma discreta predominância da prescrição apenas durante o internamento. Não foi possível estabelecer associação entre o risco trombótico individual e o tempo indicado de farmacoprofilaxia. Sabe-se que a duração da anticoagulação profilática deve ser baseada na pontuação do escore de Caprini, de modo que somente os pacientes classificados como de risco moderado (escore de Caprini 3 ou 4) devem manter profilaxia exclusivamente hospitalar^18,19^ . Embora o risco de TEV após a alta hospitalar seja amplamente reconhecido entre pacientes de alto risco, estudos demonstram que a profilaxia estendida ainda é subutilizada^12,15^ .

Este levantamento apresentou algumas limitações. O quantitativo de destinatários que receberam o questionário eletrônico não pôde ser determinado com exatidão, em virtude da sua disseminação concomitante por meios de comunicação oficiais da SBACV e pelas redes sociais dos pesquisadores, resultando na duplicação do envio do questionário para diversos profissionais. Além disso, não foram coletadas informações detalhadas sobre o perfil dos serviços onde os especialistas trabalham, mas é provável que a maneira como eles prescrevam a farmacoprofilaxia varie nas diversas regiões do país.

Mesmo com essas limitações, este levantamento inicial sobre a conduta dos angiologistas e cirurgiões vasculares atuantes no Brasil sugere a necessidade do desenvolvimento e da implementação de protocolos clínicos e diretrizes terapêuticas para prevenção em uma população que pode apresentar alto risco para a ocorrência de TEV.

## CONCLUSÃO

Este estudo revelou a heterogeneidade de condutas relacionadas à prescrição de farmacoprofilaxia em pacientes submetidos a procedimentos para amputação de MMII, especialmente quanto à ferramenta de estratificação de risco trombótico e ao tempo de manutenção da anticoagulação profilática. Ademais, a avaliação de risco de sangramento não foi incorporada pela maioria dos entrevistados. Esses achados refletem a necessidade de mais estudos para a correta segmentação desses pacientes, no intuito de dar respaldo à tomada de decisão relacionada à profilaxia nessa população.
